# The Electric Field in Solid State Nanopores Causes Dissociation of Strong Biomolecular Interactions

**DOI:** 10.1021/acs.nanolett.5c01447

**Published:** 2025-05-19

**Authors:** Wei Liu, John Andersson, Julia Järlebark, Amina Shaji, Jingjie Sha, Andreas Dahlin

**Affiliations:** † Department of Chemistry and Chemical Engineering, 11248 Chalmers University of Technology, 41296 Gothenburg, Sweden; ‡ Jiangsu Key Laboratory for Design and Manufacture of Micro-Nano Biomedical Instruments, School of Mechanical Engineering, 12579 Southeast University, Nanjing 211189, China

**Keywords:** nanopores, sensors, proteins, avidin, biotin

## Abstract

Electrical sensing with nanopores has become a widely used bioanalytical tool. However, it remains unclear if and how the extremely strong electric field generated inside the pores influences biomolecular interactions. Here we show that the field disrupts the strongest known protein–ligand interaction in biology, namely biotin–avidin bonds. Remarkably, the lifetime of the interaction is decreased by at least 4 orders of magnitude. At hundreds of mV, avidin (from egg-white) starts dissociating from biotin-functionalized nanopores over a time scale of minutes even at the maximum bond valency of four. Streptavidin-coated nanoparticles, which form many more bonds, remain bound but exhibit surface mobility due to the field. These results show that nanopore sensors can give very inaccurate results when used for affinity-based detection or biomolecular interaction analysis and that the pore environment should be regarded as potentially invasive for the molecules inside.

## Graphical abstract

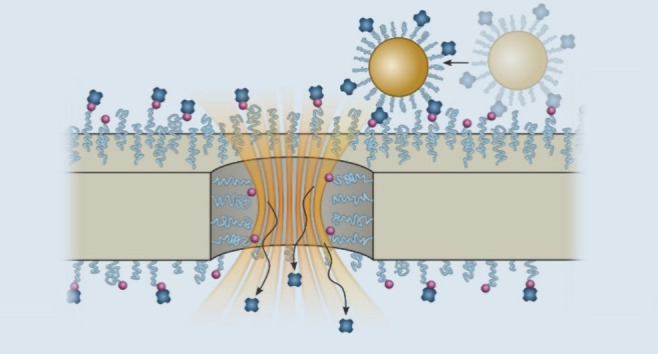

Nanopore sensors have been researched for decades and many bioanalytical applications have emerged.^1^ The technology enables label-free single-molecule detection and/or characterization by relatively simple instrumentation.^2^ Detection of biomolecular translocation or binding inside nanopores relies on changes in the ionic current from a DC voltage bias across the pore.^3^ The concept has also recently been extended to trapping of biomolecules, using docked DNA constructs and electroosmotic flow.^4^ Notably, since the nanopores are made in thin membranes (typically 20 nm or less), an extremely strong field (∼10^7^ V/m) is generated inside, even at low voltages (hundreds of mV). Such fields are around 3 orders of magnitude higher than in conventional gel electrophoresis or capillary electrophoresis^5^ and for the latter, it has still been suggested that protein denaturation can occur.^6^ For nanopores, there is evidence suggesting that proteins alter their structure during translocation.^7,8^ One can thus suspect that binding events occurring inside the pores, in particular with receptors immobilized on the walls,^3^ also will be influenced by the electrokinetic forces acting on molecules in solution phase. Importantly, this can lead to highly inaccurate results in affinity analysis or target concentration determination.

To date very few studies have considered the influence of the electric field on biomolecular interactions. Wei et al. investigated in depth how the voltage affected the residence time of histidine tags binding to nickel nitrilotriacetic acid groups on nanopore walls.^9^ However, that interaction is arguably not biological in nature and the pores used were quite special, having a conical shape and a metal coating. Freedman et al. developed a HIV antigen assay and noticed “double events” in the chronoamperometry trace which were attributed to field-induced antibody–antigen dissociation during translocation.^10^ However, there was no surface modification to restrict adsorption to the pore walls and only two voltages were tested, resulting in a relatively small effect. Finally, Kowalczyk et al. studied protein–protein interactions using chemically functionalized silicon nitride (SiN_*x*_) nanopores, but when the voltage was changed no significant effect was observed on signal characteristics such as dwell time^11^ (somewhat contradicting the previously mentioned studies). For the rest, the vast majority of papers on nanopores for affinity-based detection appear to ignore possible effects from the strong electric field.^12–20^ Clearly, for future applications of solid state nanopores, which are indeed currently focused on affinity detection^3^ and confinement,^4^ the role of the electric field needs to be elucidated.

Here we introduce nanopores functionalized with biotin and use them to capture avidin, utilizing the strongest noncovalent interaction known in biology. Nevertheless, applying voltages on the order of hundreds of mV is shown to cause release of the proteins due to electrokinetic forces. Furthermore, we investigate attachment of streptavidin-coated nanoparticles, which are more strongly bound due to the higher number of interactions. This work is important for the future of nanopore sensors since it shows that the signal transduction mechanism by itself influences biomolecules.

The biotin–avidin interaction is generally considered to be the strongest noncovalent interaction in biology, with a dissociation constant of ∼ 10^–15^ M. This is largely due to the exceptionally long lifetime of the bond^21^ (*k*_off_ < 10^–7^ s^–1^), while there are many examples of similar association rate constants.^22^ As a result, biotin–avidin bonds do not dissociate over ordinary experimental time scales and have found use in many applications.^23^ We chose this as a model system to investigate if the electric field inside nanopores can influence protein–ligand binding because: (i) the interaction is well-studied by many methods, (ii) we have established protocols for biotinylation of silica^24^ and (iii) if an effect is observed for this strong interaction, it is clear that the nanopore field will influence practically all other biomolecular interactions as well.

A requirement for precise measurements of nanopore conductance changes due to biomolecule binding/unbinding over long times (minutes or more) is that the pore diameter remains stable. Unfortunately this is normally not the case for pores in SiN_*x*_, which tend to grow in size even if no voltage is applied.^25,26^ We tested different fabrication methods and found that very stable pores could be made by electron beam lithography using a negative resist followed by reactive ion etching using inorganic films as masks (Figure 1A). To make smaller pores, we modified our previous protocol^27^ slightly. In particular, oxygen plasma was used to shrink the pillars after development. As a stability test on the final pores, we applied increasing DC potentials in 1 M KCl, which led to insignificant or very small (1–2%) increments in pore diameter over ∼ 1 h (Figure 1B). We also tested simpler fabrication approaches with a positive resist^28^ or controlled dielectric breakdown,^29^ but both resulted in pores and membranes that were generally much less stable. Note, however, that the stable pores could not be made smaller than 30–40 nm, at least not while maintaining a reproducible circular shape (Figure 1C). This prevented detection of single avidin molecules^4^ by resistive pulses, but the high stability enabled quantitative analysis of surface binding (Figures S1–S2).

**Figure 1:**
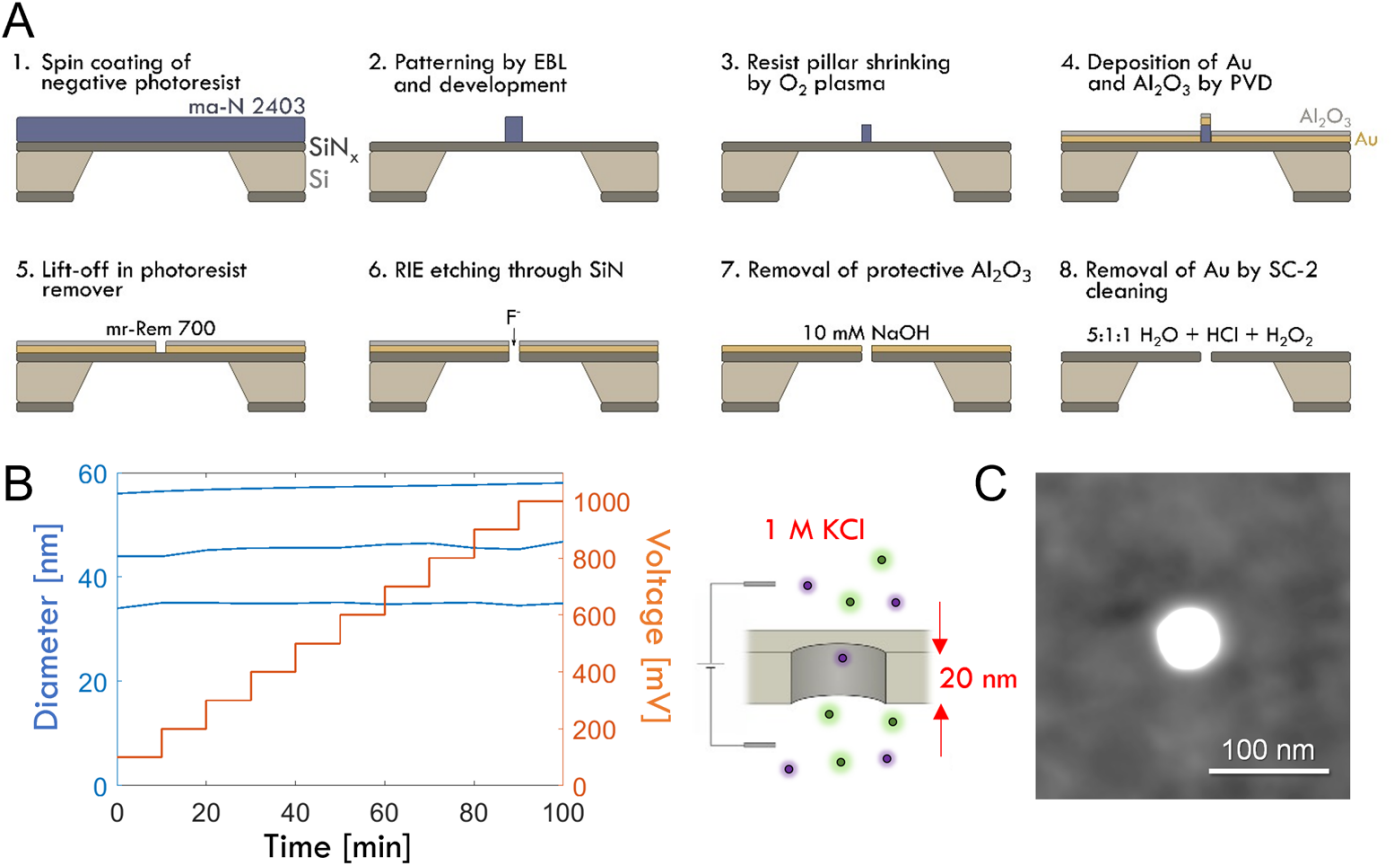
Fabrication of ultrastable nanopores in 20 nm SiN_*x*_. (A) Electron beam lithography process with negative resist. (B) Stability tests with increasing DC voltage. The pore diameter was monitored by quickly measuring the conductance before each new voltage was applied. (C) Electron microscopy image (transmission mode) of a pore with the gold film still remaining.

We modified the SiN_*x*_ nanopores with poly­(ethylene glycol) (PEG) brushes as described previously, creating a strongly antifouling layer.^24^ The normalized conductance drop after the PEG conjugation is shown in Figure 2A for different pore diameters. Since the brushes are thinner (10 nm) than the pore radius, the effect on pore conductance is higher for smaller pores. Here it should be noted that the hydrophilic polymer brushes allow small species like ions to pass.^30^ We simulated the expected conductance change assuming a 1.6 nm solid grafting layer^24^ beneath a uniform PEG brush and found good agreement (Figure 2A) when the conductivity of the polymer zone was set to 50% of the bulk medium. Based on the volume fraction of polymer inside the brush,^24^ this value is in fair agreement with bulk conductivity changes measured for electrolytes containing PEG.^31^ As further control, the simulated bare pore conductance was in agreement with the established analytical model to calculate pore diameter^29^ (Figure S3).

**Figure 2:**
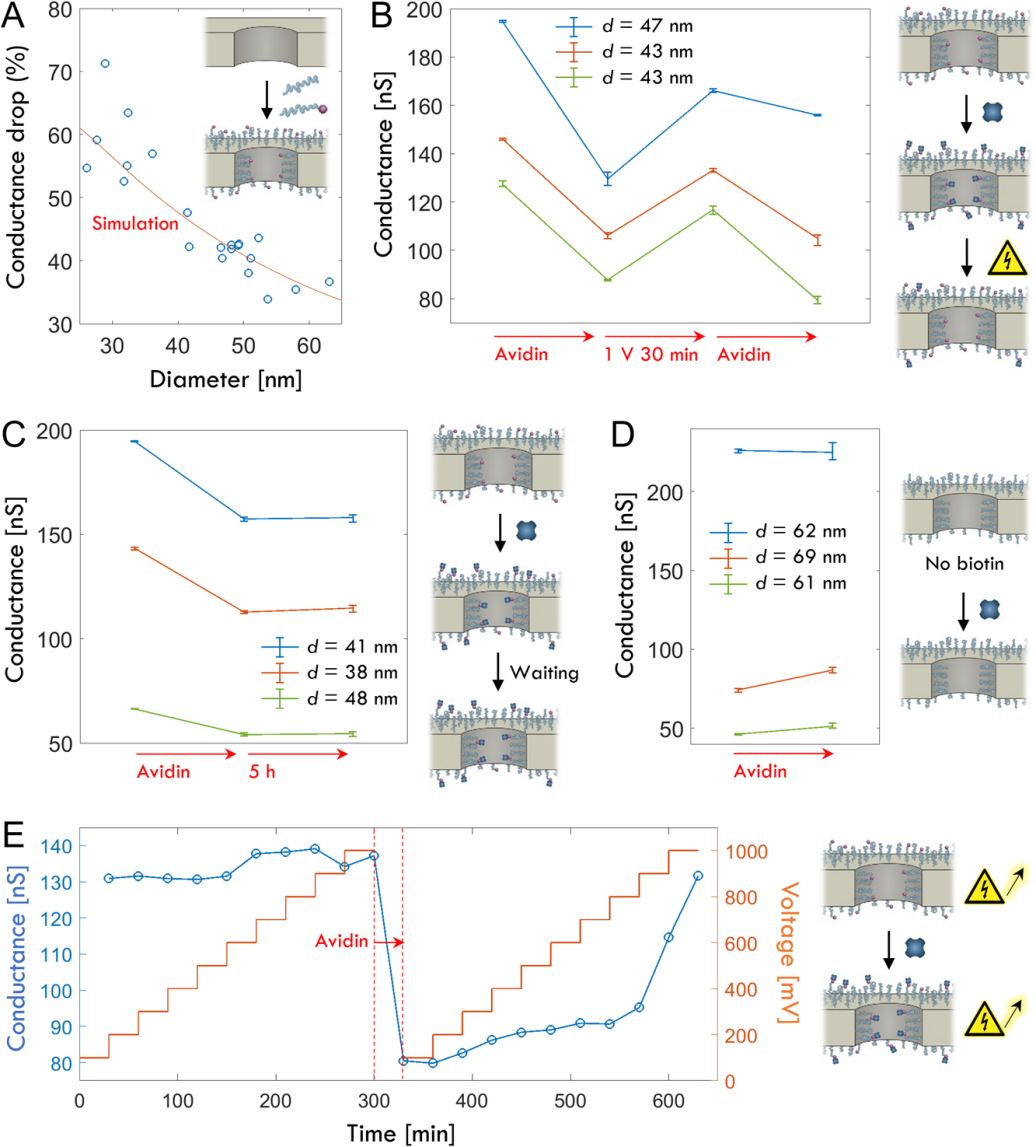
Breaking the biotin–avidin interaction by the electric field inside a nanopore. (A) Relative conductance change of individual pores after silanization and conjugation of PEG chains. Results from simulations are also plotted, using a conductivity for the brush which is 50% of the bulk medium (1 M KCl). (B) Conductance changes after avidin binding and the application of 1 V DC bias for 30 min. Data are shown for three different bare pore diameters. Error bars represent variation from repeated voltage sweeps to determine conductance. (C) Equivalent data but without applying a voltage. No significant conductance increase is observed. (D) Equivalent data but on pores without any biotinylated PEG chains. No significant conductance decrease is observed. (E) Conductance changes during gradual increase in voltage before and after avidin binding. The conductance is quickly measured before every new voltage is applied.

To study field-induced dissociation of biotin–avidin bonds, we included 17% molar fraction of PEG chains with biotinylated end groups. For ∼ 2 kg/mol PEG, this corresponds to 0.087 biotins per nm^2^.^24^ The pores were exposed to avidin (100 μg/mL) for 30 min and excess proteins in solution were then washed away. This led to a saturated binding of 250 ng/cm^2^ on planar surfaces (Figure S4). Figure 2B shows typical signals from avidin binding and from subsequently applying 1 V for 30 min. The latter lead to a clear increase in conductance. Upon addition of avidin a second time, a conductance decrease was once more observed. As further controls, we also verified that there was no spontaneous dissociation (after >5 h) if no voltage was applied (Figure 2C) and that there was no signal from avidin in the absence of biotin as expected^24^ (Figure 2D). These results prove qualitatively that applying a voltage induces avidin dissociation. Furthermore, a gradual conductance increase with increasing voltage was observed after avidin binding, noticeable already at 200 mV (Figure 2E). The reason why the conductance is not directly recovered after one voltage step is partly because the release is a stochastic process, but also because the dissociation will be easier at high-field regions in the nanostructure. Some proteins will on average require a higher voltage for the release to occur and those bound away from the pore will not be released at all, nor will they contribute to the signal (Figure S5).

To understand the mechanism of protein dissociation, both electroosmotic and electrophoretic forces need to be considered. SiN_*x*_ is normally negatively charged and avidin is positive, which means that close to physiological pH, the forces are expected to be aligned.^32^ However, here the surface is chemically modified such that it is expected to be more neutral,^24^ which means that electroosmotic forces should be small. To confirm this, we measured zeta potentials (ζ) during the chemical modification process by streaming currents on sandwiched SiN_*x*_ films identical to the membranes used for making pores (Figure 3A). At 1 mM salt, the bare SiN_*x*_ is negative at all pH tested and becomes more positive after silanization, then closer to neutral after the remaining steps. The surface becomes more positively charged after avidin binding due to its high isoelectric point^32^ (pI ≈ 10). Still, negative charges dominate at high pH, which is in agreement with previous studies and attributed to chemical interactions with hydroxyl ions.^33^ At pH 8, as the ionic strength is increased, the zeta potential becomes very low in magnitude for surfaces with PEG and avidin. The electric field magnitude *E* is simulated for different pore diameters in Figure 3B, including the 10 nm PEG coating with a 50% reduced conductivity. Taking the field to be *E* = 10^7^ V/m inside the pore and assuming |*ζ*| ≈ 2 mV indicated by data in Figure 3A, the electroosmotic flow velocity can be estimated^32^ as *v* = *ζεE*/*η* ≈ 1 cm/s (*η* = 10^–3^ Pas, *ε* = 80*ε*_0_). Detailed simulations with literature values of SiN_*x*_ surface charge density^34^ gave similar values (Figure S6). The drag force acting on the protein is *F* = *vk*_B_ *T/D* with diffusivity *D* = 6 × 10^–7^ cm^2^/s according to Spinke et al.^35^ Inserting all values gives *F* < 1 pN due to electroosmosis, a relatively weak force.

**Figure 3:**
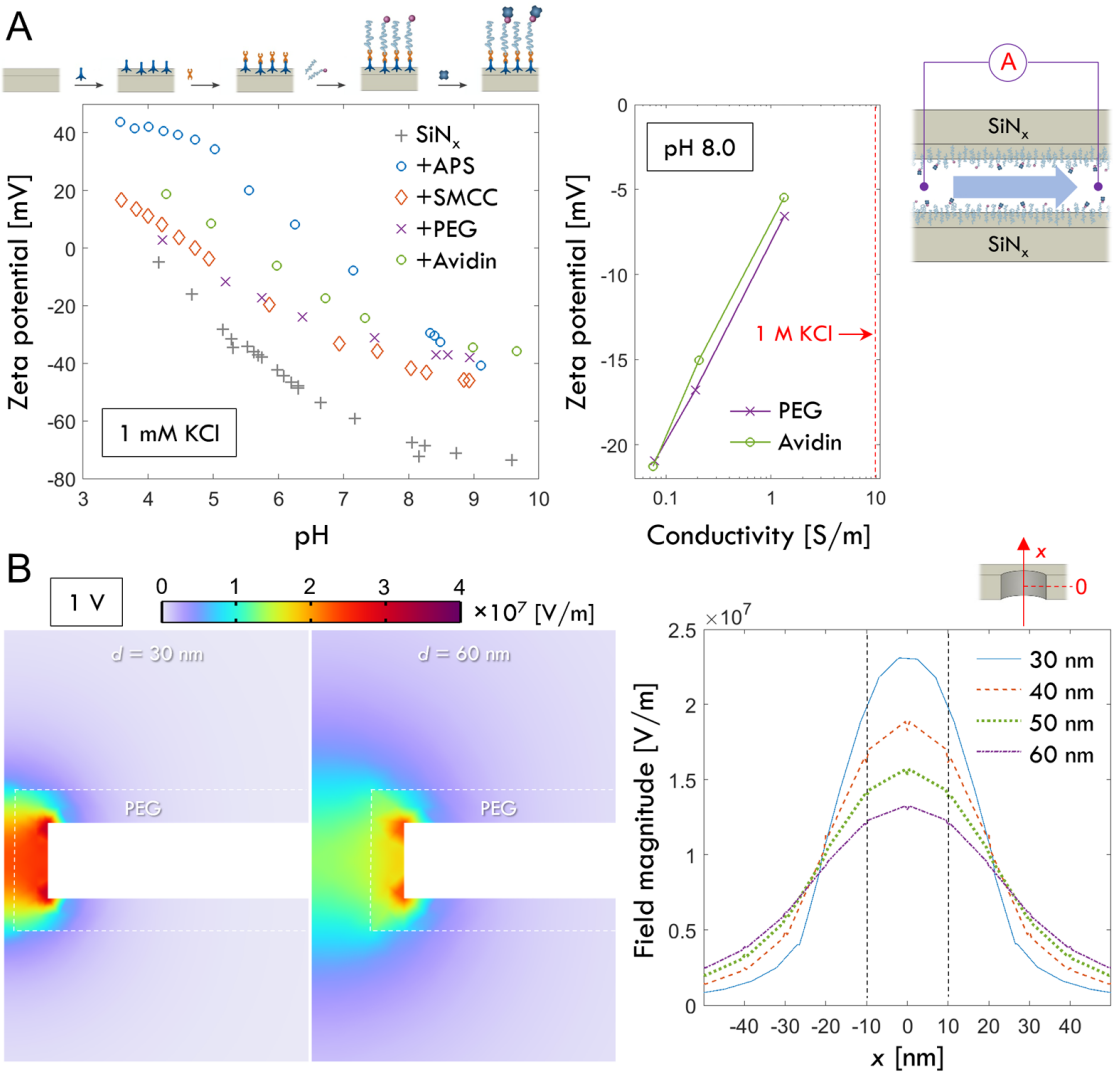
Elucidating the electrokinetic effects. (A) Zeta potentials vs pH at low salt measured for all steps in the chemical modification of silicon nitride. The protocol was presented by us previously^24^ (but in that study no zeta potentials were measured). APS stands for aminopropylsilatrane, and SMCC stands for sulfosuccinimidyl 4-(*N*-maleimidomethyl)­cyclohexane-1-carboxylate. The right plot shows zeta potentials after PEG-biotin and avidin binding at pH 8 for increasing salt content. (B) Simulated field distribution in a 30 nm and a 60 nm nanopore with PEG-biotin. The field along the central axis is also plotted for four different pore diameters, showing a higher field for smaller pores.

In comparison, the electrophoretic force is directly obtained as the product of the field and the charge *Q* = *ne*, where *e* is the elementary charge and *n* is the net charge valency. Avidin does not have a surplus of basic residues but it is still net-positively charged due to glycosylation with glucosamine groups.^36^ Although we cannot know the ionization state of the protein during the experiments, even a *single* positive charge will generate an electrophoretic force >1 pN at 10^7^ V/m. Hence, we conclude that the electrophoretic force is dominating. Still, it should be noted that both forces are aligned. Higher “rupture forces” in the range 10–100 pN have been observed for biotin–avidin with atomic force microscopy^37,38^ or optical tweezers.^39^ However, our forces cannot be compared to those in such experiments since the load is then increased rapidly until rupture is enforced, typically in <1 s. This is different from our nanopores, where the proteins are exposed to a steady force defined by the voltage. To rule out other effects, we also simulated the temperature increase in the pore, which was less than 1 K at 1 V even without taking convective cooling into account (Figure S7). This is in agreement with experimental work showing that considerably larger pores and higher voltages are needed for significant heating.^40^

Next, we consider the change in the dissociation constant *k*_off_ caused by the nanopore field quantitatively. A single biotin–avidin bond takes on the order of 1/*k*_off_ ≈ 10^7^ s to spontaneously dissociate, while in our case most proteins dissociate in ∼ 1000 s. However, each avidin is a tetramer with four binding sites. The molar surface coverage was determined to be almost exactly 25% (0.0224 nm^–2^) of that for biotin (Figure S4), which means there are four bonds per protein (since otherwise more avidin would bind). This is due to the flexibility of the PEG chains, which will occupy free binding sites faster than new proteins arrive during binding.^41^ Hence, the reduction of the lifetime of the interaction will be *larger* in case of a single biotin–avidin bond. In addition, increasing the higher ionic strength (we use 1 M KCl as standard in nanopore sensing) is known to lead to an *increased* affinity.^42^

The bond rupture events can also be analyzed from a free energy perspective. Since our experiments are done without any avidin in solution, the equilibrium state is for all proteins to dissociate, but this is prevented by a considerable barrier ΔG* which can be related to *k*_off_ by Arrhenius expressions. When the protein is exposed to a steady force *F*, ΔG* will be reduced by *Fz*, where *z* is the characteristic distance that the ligand needs to move until the transition state is reached.^43^ Given that the electrophoretic force is dominating, we can estimate the effect on the dissociation rate constant as

1 $$k_{\mathrm{off}}\left(U\right)=k_{\mathrm{off}}\left(U=0\right)\times \exp\left(\frac{Unez}{{hk}_{B}T}\right)$$

Here the field is approximated as *E* = *U*/*h* where *h* is the pore length (membrane thickness) and the exponential is essentially representing ΔΔG* = *Fz* normalized by *k*_B_ *T*. The model can be used to estimate the effect of the nanopore field if the ionization state of the molecule in solution phase is known, as discussed by Wei et al.^9^ The distance *z* should be approximately the depth of the binding socket. For the case of avidin, the binding pocket matches biotin in size^44^ and the length of biotin is ∼ 0.9 nm. Notably, if the relative change in *k*_off_ is known experimentally, it is possible to calculate *n* (an integer) with quite high precision due to the exponential dependence. We obtain *n* = 6 for avidin for a change in *k*_off_ by a factor of ∼ 10^4^, but since each protein actually has four bonds the model should ideally be extended to be fully valid.^37^

A higher number of bonds is expected to cause a major increase in affinity, i.e. the effect from avidity.^45^ To investigate this we introduced streptavidin-coated gold nanoparticles to the biotinylated nanopores. The binding kinetics of 15 nm core particles in 1 M KCl were also characterized by quartz crystal microbalance with dissipation monitoring (Figure 4A), showing a saturated response of up to 100 Hz at the fundamental resonance and no spontaneous dissociation after rinsing. Considering the gold mass as dominating and using the Sauerbrey constant (17.7 ngcm^–2^Hz^–1^) as an approximation, the response corresponds well to a monolayer with fractional coverage in the range 20–40%. After exposing the biotin-functionalized nanopores to the same particle concentration for the same duration, a decrease in conductance could be detected. However, the signals were sometimes very low, especially for the smaller pores, suggesting that most particles were bound at the edge of the pore opening. This is in agreement with previous studies on other nanopore structures and can be explained by the fact that the particles become immobile immediately after attaching to the surface,^46^ making it hard for them to reach the pore interior. (Note that the voltage was off during binding.) Complementary simulations confirmed that the conductance change from a 15 nm particle binding outside of the pore was very small (Figure S8). Nevertheless, we could see signals from particles in several experiments (Figure 4B). In these cases, after applying a voltage to attempt release (1 V for 30 min), there was never a significant increase in conductance, which shows that the particles remain. The increased avidity leads to a stronger attachment and this effect dominates over the increased electrokinetic forces acting on a particle (as compared to a protein). The zeta potential of the particles was negative by a few mV and the electrophoretic force is expected to scale linearly with surface area since this is where the charged groups are exposed to the field. The maximum number of interactions is also expected to be proportional to particle surface area. However, due to configurational bond entropy, the avidity increases very rapidly in a nonlinear manner with the number of interactions,^45^ especially when the receptors are tethered to flexible chains such as PEG. This explains why the particles are not released as easily as the individual proteins, even if stronger electrokinetic forces act on them. Note that for streptavidin, the affinity to biotin is weaker, but still very strong in absolute numbers (*k*_off_ ≈ 3 × 10^–6^ s^–1^).^47^

**Figure 4:**
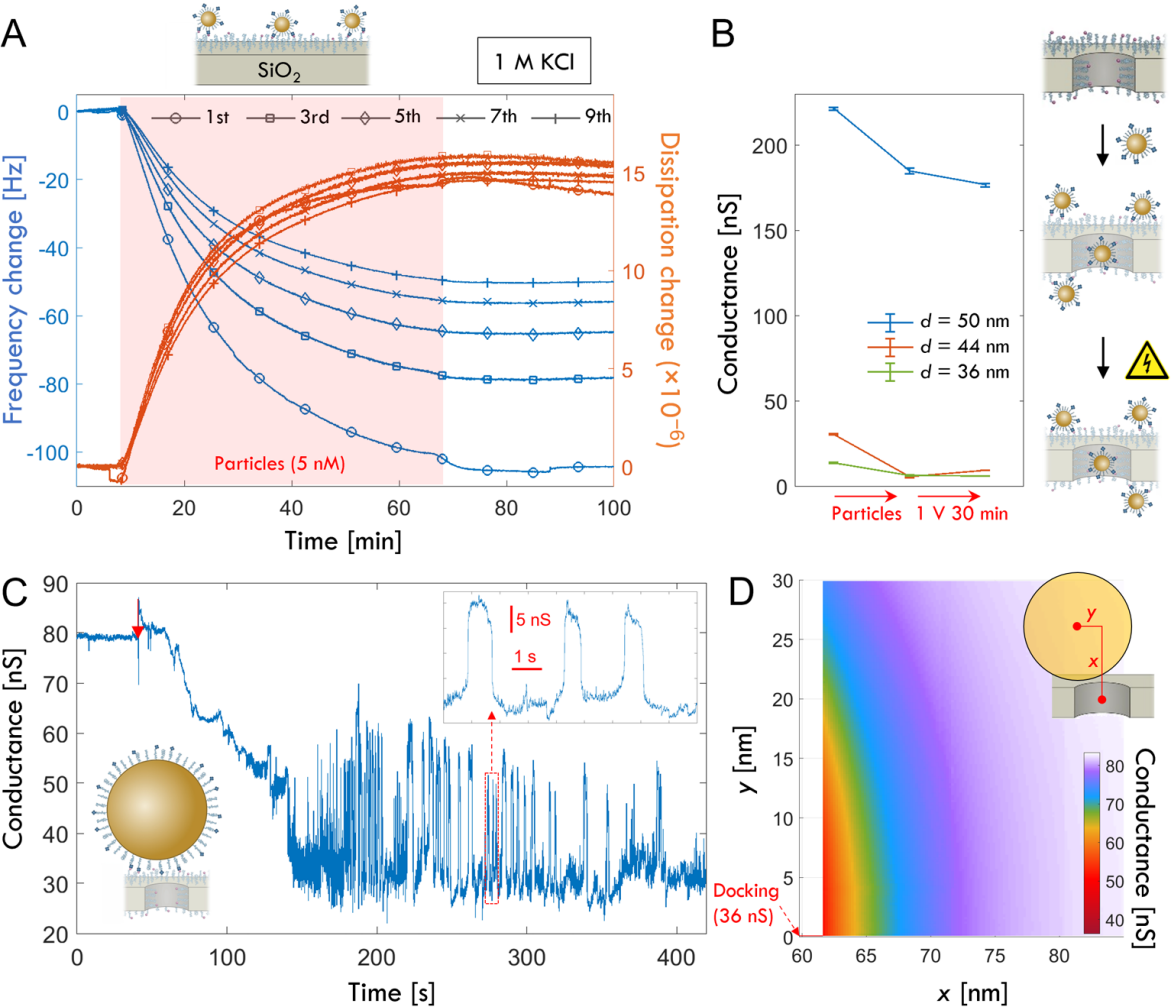
Streptavidin-coated gold nanoparticles are not released by the field but show surface movement. (A) Binding of streptavidin-coated gold nanoparticles to a silica surface functionalized with biotin-PEG analyzed by quartz crystal microbalance with dissipation monitoring. The measurement was performed in 1 M KCl, and 15 nm particles were introduced at 5 nM. (B) Conductance decrease after binding of 15 nm particles and lack of a significant conductance increase after applying 1 V for 30 min (three different pores). (C) Real-time chronoamperometric detection of attachment of a larger (100 nm) particle to a 31 nm pore using 700 mV bias. A gradual conductance decrease and fluctuations can be observed after the initial particle attachment, which is assumed to occur at the arrow marker. (D) Simulation of conductance depending on the position of the particle above the pore. The *x* coordinate is the distance from the pore center to the particle center, and the *y* coordinate is the radial displacement. The red line indicates the “docked” position where *y* = 0 and *x* approaches the minimum possible value considering solid particle and pore sizes.

Even if the particles are not released, it is expected that the strong forces will cause biotin–avidin bonds to be broken and reformed dynamically, which should enable mobility. To investigate this aspect, we detected binding of nanoparticles to the pores in real-time by chronoamperometry. Figure 4C shows the attachment of a larger (100 nm core) particle to a pore using a voltage of 700 mV. Particles were introduced on the side with negative polarity such that the field guides them toward the pore. Interestingly, the particle docking did not appear as an immediate event. Instead, a gradual conductance decrease was observed over ∼ 1 min, suggesting that the particle was moving toward the pore relatively slowly. For comparison, when DNA constructs dock onto bare SiN_*x*_ nanopores, the current instantly goes from the baseline to a new constant value.^48^ However, DNA constructs are then electrostatically repelled by a bare SiN_*x*_ surface, while in our case we have particles that form bonds with a functionalized surface. Therefore, a slow movement along the surface toward the pore center can be expected, given that the particles maintain mobility. Normally, streptavidin-coated particles bound to biotinylated surfaces are only mobile if the receptor layer is fluid.^49^ However, in our case, the particle is exposed to the nanopore field. Hence, our interpretation is that the electrokinetic forces cause surface movement toward the pore to minimize the electric potential energy. After particle binding, the conductance eventually reaches values below 40 nS, indicating that the particle ends up very tightly attached to the pore opening. Additionally, large fluctuations can be seen, strongly suggesting dynamic changes in the precise position of the particle. We confirmed this by simulating the conductance blockade for a particle above a pore (Figure 4D). Agreement with the lowest experimental conductance was only achieved with some compression of the PEG coating. The magnitude of the fluctuations is consistent with movement up to ∼ 10 nm radially and/or a few nm along the pore axis.^48^ Hence, the particles are not released but seem to exhibit surface mobility.

In conclusion, we have shown that the field inside solid state nanopores dissociates biotin–avidin bonds, the strongest known protein–ligand interaction. We point out once more the consequences of these findings: if nanopore sensors are used for affinity-based detection or analysis, results may be inaccurate by many orders of magnitude unless the effect is properly accounted for. This applies in particular when surface-immobilized receptors are used as one of the molecules is then stuck, but the strong field may also influence complexes in solution phase.^4^ In particular, oppositely charged species will be pulled in opposite directions. Dissociation^10^ or structural changes^7,8^ may potentially occur even during translocation, especially in combination with surface interactions.^50^

## Supplementary Material



## Notes

Experimental details, example of voltage–current sweeps, example of ion current traces, simulations of bare pores, complementary SPR data, simulations of conductance change from avidin, electroosmotic flow, temperature increase and conductance change for particle binding (PDF)
