# Supplementary material for: The Electric Field in Solid State Nanopores Causes Dissociation of Strong Biomolecular Interactions
Source: Nano Lett. 2025 May 19;25(24):9654–61. doi: 10.1021/acs.nanolett.5c01447 (PMC12186603; doi:10.1021/acs.nanolett.5c01447)
Supplement: Supplementary file 1 [file nl5c01447_si_001.pdf]

The Electric Field in Solid State Nanopores Causes Dissociation of Strong Biomolecular Interactions

Wei Liu,[2] John Andersson,[1] Julia Järlebark,[1] Amina Shaji,[1] Jingjie Sha[2] and Andreas Dahlin.[1]

1 Department of Chemistry and Chemical Engineering, Chalmers University of Technology, 41296 Gothenburg, Sweden.

2 Jiangsu Key Laboratory for Design and Manufacture of Micro-Nano Biomedical Instruments & School of Mechanical Engineering, Southeast University, Nanjing 211189, China.

Experimental

Chemicals: All measurements were performed with tris buffer at pH 8.0 with 1 M KCl as electrolyte unless otherwise stated. For making aqueous solutions, milliQ water (18.2 MΩcm) was used. Ethanol (99.5%) was purchased from Solveco. H_2O_2 (35%) was purchased from Scharlau. Sulfosuccinimidyl 4-(N-maleimidomethyl)cyclohexane-1-carboxylate (sulfo-SMCC) was purchased from Thermo Fischer Scientific. Thiol-PEG-methyl-ether 1.8 kg/mol and biotinylated version (2.2 kg/mol) were purchased from Laysan Bio Inc. Avidin (from egg white, ≥ 98 %) KCl, Na_2SO_4 and phosphate buffered saline (PBS) tablets were purchased from Sigma Aldrich. The streptavidin-functionalized gold nanoparticles were purchased from Luna Nanotech and had a zeta potential of −2 mV according to the manufacturer.

Nanofabrication: Electron beam lithography was performed as described previously[1] with some modifications to reduce pore diameter: Baking for 10 min at 180 °C was performed before resist spinning and for 10 min at 100 °C afterwards. 70 s O_2 plasma etching at 50 W was used to shrink the pillars after resist development. Increasing the etching time further leads to collapse of the majority of resist pillars due to the large aspect ratio. To obtain a pore in a pure silicon nitride membranes, the gold film was removed by SC-2 cleaning (HCl : H_2O_2 : H_2O at 1:1:5 volume ratios at 80 °C for 30 min). The final chips (1×1 cm^2) had one pore each on the 40×40 μm^2 membrane with 20 nm thickness as determined by spectroscopic ellipsometry.

Conductance measurements: Nanopore conductance and chronoamperometry was measured by an Axopatch 200B (Molecular Devices). Liquid cells from Norcada were used and placed in a Faraday cage from Northern Nanopore. All diameters stated (*d* values) refer to bare pores before chemical modification and determined from the conductance.

PEG modification: Samples were cleaned with hot piranha (conc. H_2SO_4 and 30 % H_2O_2 mixed in volume ratio 3:1 for 20 min). The nanopores were modified similarly to the *ex situ* protocol published previously.[2] A droplet of APS (460 µM in 99.5 % EtOH) was placed on the chip for 1 min followed by rinsing in EtOH and drying. The chip was then placed on a hotplate set to 125 °C for 1 min, rinsed with water and dried. Next, sulfo-SMCC at 0.5 g/L in 10× diluted PBS buffer was placed on the sample for 1 min. PEG was introduced at 1 g/L in 0.9 M Na_2SO_4 for 2 h. Pores where the conductance decrease after PEG grafting was clearly too high (~80% or more) or too low (typically < 30%) were excluded from further experiments. This can occur

---

[1] Malekian et al. *Nanoscale Advances* **2019**, 1, 4282-4289.

[2] Andersson et al. *ACS Applied Materials & Interfaces* **2023**, 15, 10228-10239.

when the silanization or crosslinker binding leads to multilayer formation or when the layers do not form properly due to impurities etc. (The majority of the pores did not exhibit such behavior.)

Zeta potential measurements: Streaming current measurements were performed using a zeta potential analyzer ZPA 20 with a liquid dosing unit LDU 25 (DataPhysics Instruments Gmbh). A MC-ZPA/S flow cell for 20×10 mm samples was used and all measurements were performed in bidirectional (oscillating flow) mode approximately in the interval 600-1400 mbar (absolute pressure) with 100 μm gap height, piston amplitudes set to 1-2 mm and frequency of 0.3 Hz. The instrument automatically measures pH during titration and adjusts with HCl/NaOH. A new volume of 250 mL was introduced when changing salt content and the conductivities were measured by the instrument.

QCMD measurements: Experiments were performed with a Q-Sense E4 (Biolin Scientific) using SiO_2 coated sensor crystals treated exactly like the nanopore chips but without piranha cleaning. The temperature was set to 25 °C and the flow rate was 150 μL/min.

Simulations: COMSOL Multiphysics was used for calculating pore conductance using 3D models. Boundaries were introduced 500 nm away from the pore vertically and laterally. The 10 nm PEG coating was included in all simulations unless otherwise stated, with a 1.6 nm grafting layer underneath. Particles and proteins (with zero conductivity) were allowed to penetrate the soft PEG (which had a conductivity set to 50% of the bulk conductivity) but not the solid grafting layer (zero conductivity). Temperature was simulated by assigning further properties to the materials. For SiN_x: density 3100 kg/m^3, specific heat capacity 700 Jkg^{-1}K^{-1} and thermal conductivity 3.2 Wm^{-1}K^{-1}. The 1.6 nm grafting layer was assumed to have the same properties as SiN_x. The electrolyte and the PEG were assumed to have the same thermal properties as water. The temperature at the boundaries was set to 293.15 K. Electroosmotic flow was simulated in 2D (cylindrical symmetry) by combining the Poisson-Boltzmann equation, the Navier-Stokes equation and the Nernst-Plank equation. The surface charge density of the SiN_x was set to −0.02 C/m^2. The relative permittivity for the liquid was set to 80 and the dynamic viscosity to 10^{-3} Pas. The diffusivities of K^+ and Cl^- were set to 1.957×10^{-9} m^2/s and 2.03×10^{-9} m^2/s respectively.

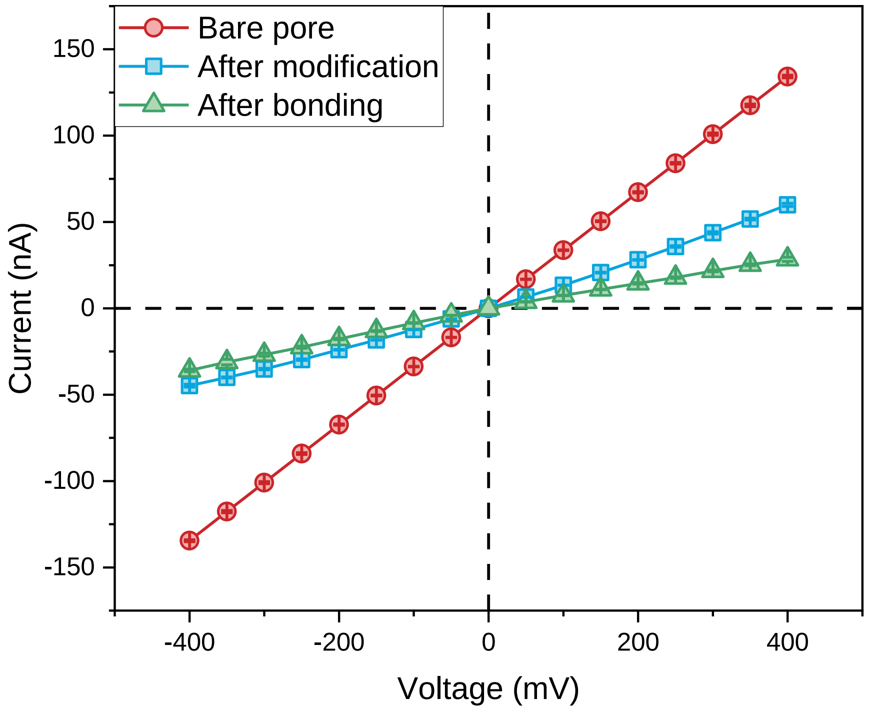

Figure S1 Typical IV curves for detecting molecular binding to the pore surface, i.e. PEG modification and avidin binding. The conductance is determined from the linear fit.

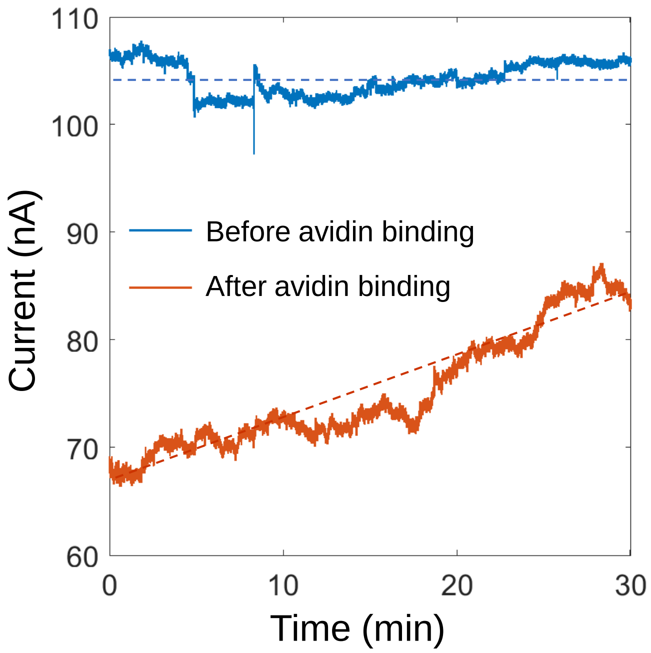

Figure S2 Example of current traces while applying a voltage for release, in this case 800 mV for 30 min. Note that over such a long time there are always some random fluctuations and disturbances appearing in the data. This can be attributed to, for instance, the formation of bubbles. While some of the sudden changes in the red trace could tentatively be due to single molecule release events, this cannot be concluded since similar steps appear also in the blue control trace. Also, the expected signal from a single molecule is ~1 nS at the most, based on simulations (Figure S5), which is similar to the short-term noise. However, the current clearly increases slowly with time when avidin has been bound, while it remains constant on average in the control.

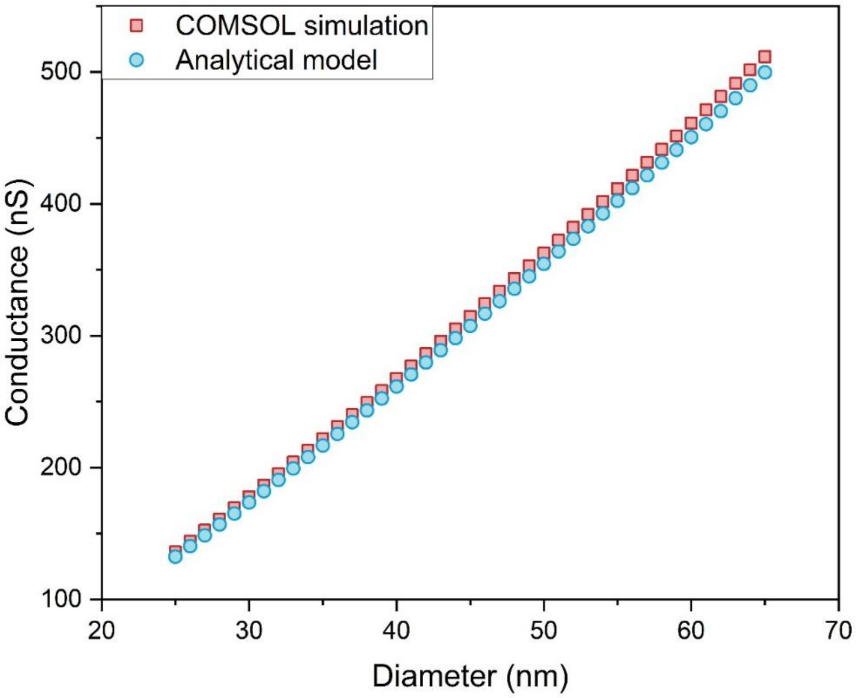

Figure S3 Agreement between COMSOL simulations and the established analytical model for relating conductance to diameter for bare pores, i.e. $G = g[4h/[\pi d^2] + 1/d]^{-1}$ where g is the bulk medium conductivity (g = 10.7 S/m for 1 M KCl).[3] The membrane thickness was always h = 20 nm. The seemingly linear relation is because access resistance dominates over the resistance of the cylindrical pore.

---

[3] Jarlebark et al. *Analytical Chemistry* **2025**, 97, 4359-4364.

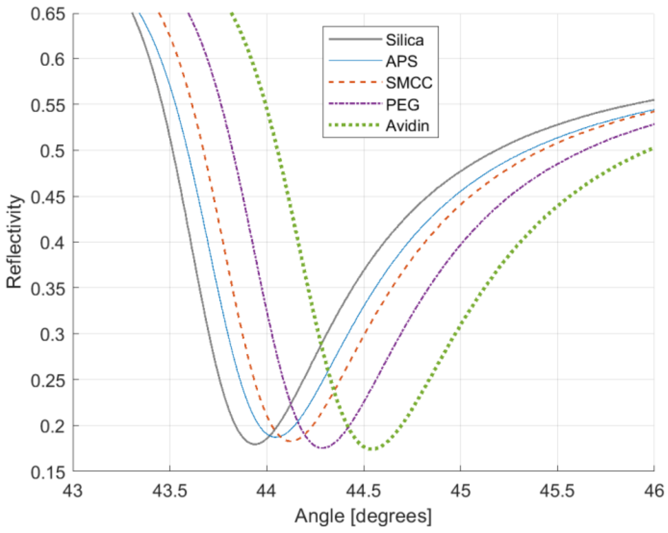

Layer	[nm]	n	k
Prism	∞	1.52	0
Cr	2	3.53	4.27
Au	50	0.22	3.93
SiO_2	10.89	1.46	0
APS	0.78	1.42	0
SMCC	1.03	1.42	0
PEG/PEG-biotin	1.40	1.46	0
Avidin	1.85	1.53	0
Bulk	∞	1.00	0

Figure S4 Complementary surface plasmon resonance data (at 670 nm) of avidin binding and (lack of) release in 1 M KCl. Spectra were measured in air after each modification step, including deposition of the silica film by atomic layer deposition. The table shows the thickness of each layer, determined by fitting to the corresponding spectrum using a known refractive index. The molecular weights of the PEG chains and of the protein (67 kg/mol) were then used to determine surface coverages in terms of molecules per area. In addition, injection of biotinylated 20 kg/mol PEG to a surface saturated with avidin gave a very small response (< 0.01° during real-time monitoring), further proving that the bond valency is maximal for almost all proteins.

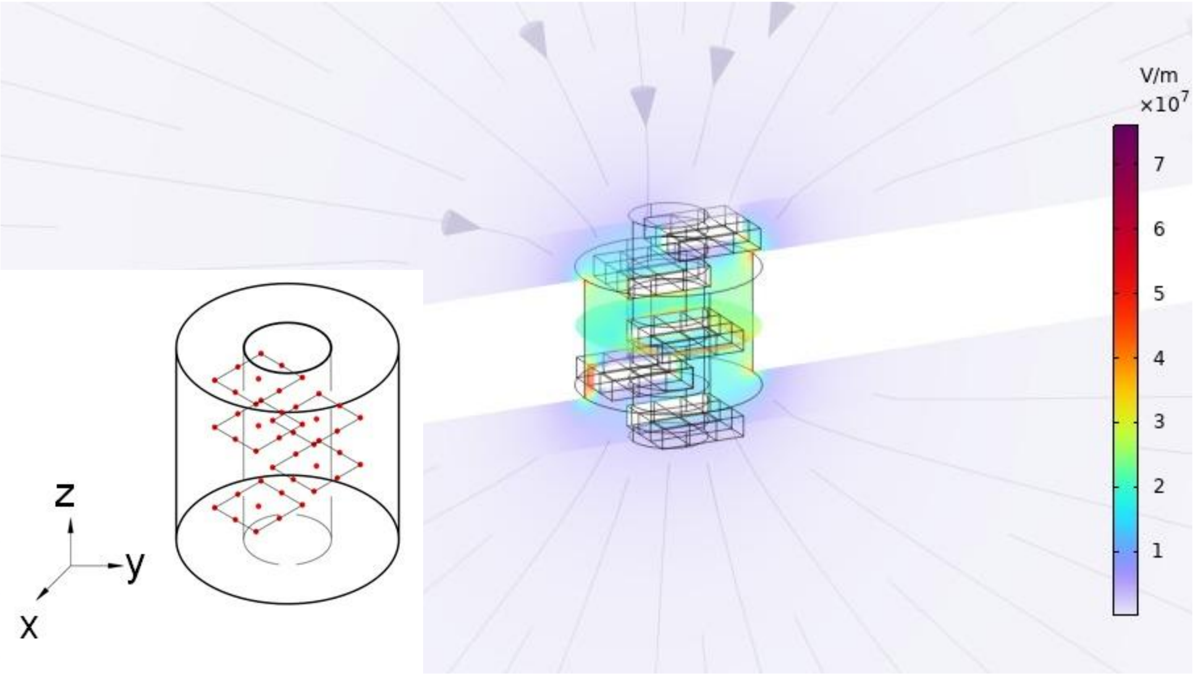

Figure S5 Simulated conductance change from avidin binding. 45 avidin molecules were placed inside a nanopore with 38 nm bare diameter as shown in the image. This resulted in a conductance change from 126 to 92 nS, in good agreement with experiments. Based on the results which provided the surface coverage (Figure S4), there should be ~50 avidin molecules inside a pore of this size (ignoring curvature effects). If avidins were placed also outside the pore, the conductance change was an additional 2 nS, showing that the majority of the signal originates from binding to biotin in the pore interior. The proteins were represented as rectangular boxes of size 56×50×40 $Å^3$ based on the crystal structure of avidin.[4]

[4] Rosano et al. *Biomolecular Engineering* **1999**, 16, 5-12.

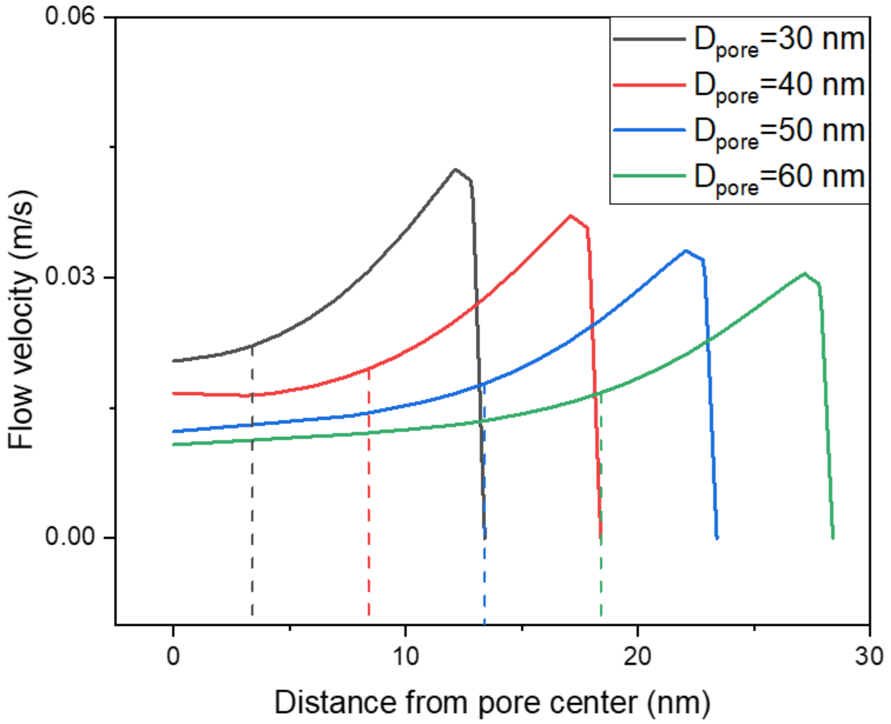

Figure S6 Simulation of electroosmotic flow velocity profile. The expected PEG brush boundary is indicated by dashed lines. It is assumed that the polymer does not hinder the flow, which means that the values are likely to be overestimated. The bias voltage was 1 V.

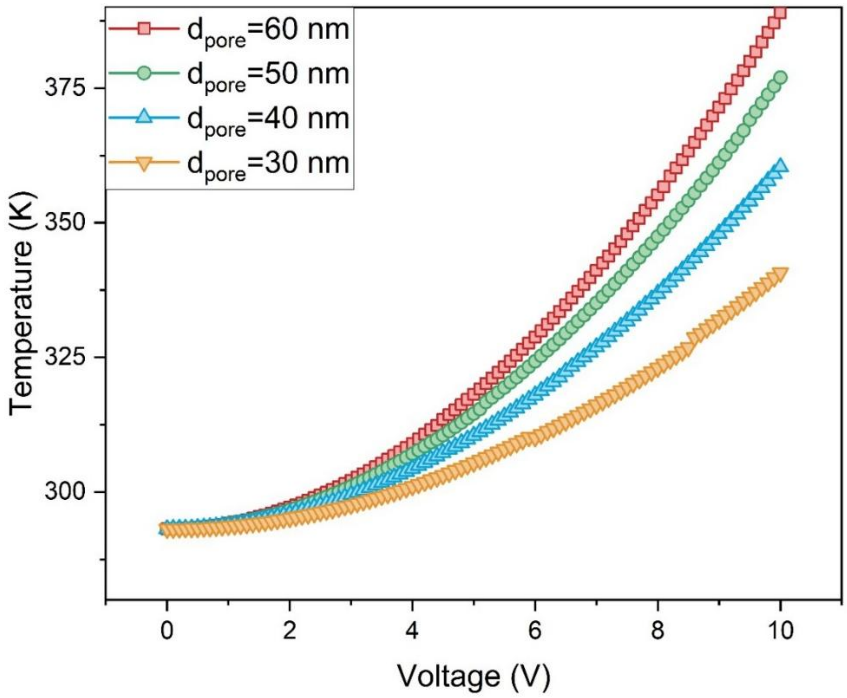

Figure S7 Simulations of temperature increase inside the pore. The steady state temperature is increasing with the square of the voltage as expected, but up to 1 V the increment is only ~1 K for all pore diameters tested.

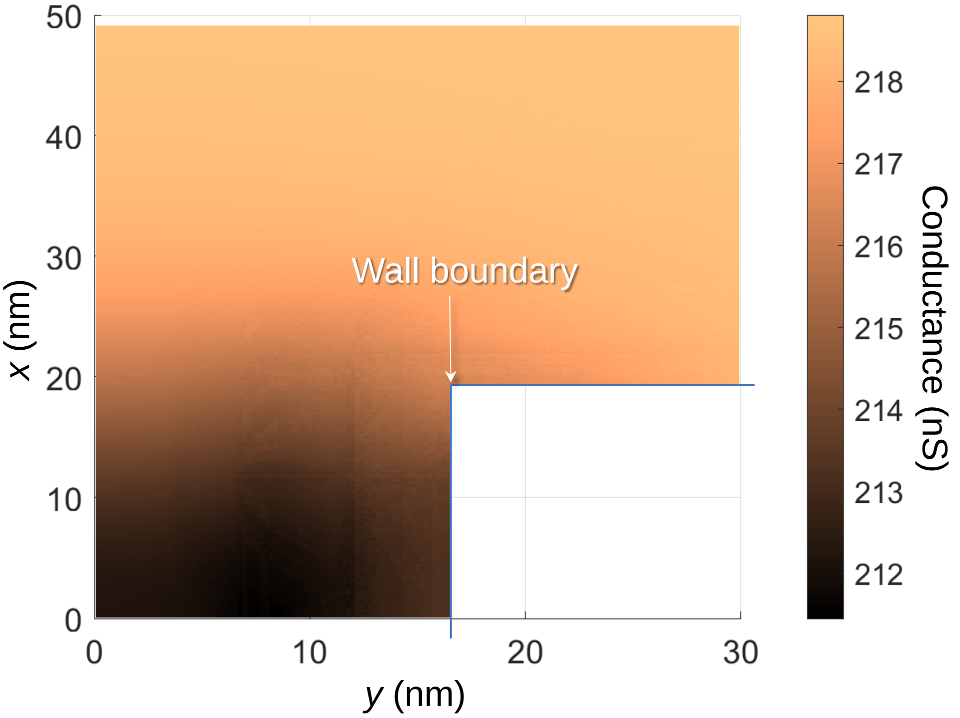

Figure S8 Simulations of pore conductance after attachment of a 15 nm particle depending on its position. The bare pore diameter is 51 nm. Even in the center of the pore ($x = y = 0$) the conductance change is < 10 nS. This explains why the signals from particle binding were generally low.
